# Predicted Cognitive Conversion in Guiding Early Decision-Tailoring on Patients With Cognitive Impairment

**DOI:** 10.3389/fnagi.2021.813923

**Published:** 2022-02-02

**Authors:** Yu Zheng, Yin Liu, Jiawen Wu, Yi Xie, Siyu Yang, Wanting Li, Huaiqing Sun, Qing He, Ting Wu

**Affiliations:** ^1^Department of Rehabilitation Medicine, The First Affiliated Hospital of Nanjing Medical University, Nanjing, China; ^2^Division of Brain Rehabilitation, Department of Neurology, The First Affiliated Hospital of Nanjing Medical University, Nanjing, China; ^3^Department of Neurology, The First Affiliated Hospital of Nanjing Medical University, Nanjing, China; ^4^Intensive Care Unit, Wuxi No.2 People’s Hospital, Wuxi, China; ^5^Department of Neurology, Xuzhou First People’s Hospital, Xuzhou, China; ^6^Department of Neurology, The Affiliated Hospital of China University of Mining and Technology, Xuzhou, China

**Keywords:** Alzheimer’s disease, mild cognitive impairment, decision-tailoring, deep learning, Alzheimer’s disease assessment scale, cognitive conversion, medical treatment reassignment

## Abstract

**Background:**

Cognitive decline is the most dominant and patient-oriented symptom during the development of Alzheimer’s disease (AD) and mild cognitive impairment (MCI). This study was designed to test the feasibility of hybrid convolutional neural networks and long-short-term memory (CNN-LSTM) modeling driven early decision-tailoring with the predicted long-term cognitive conversion in AD and MCI.

**Methods:**

Characteristics of patients with AD or MCI covering demographic features, clinical features, and time-dependent neuropsychological-related features were fused into the hybrid CNN-LSTM modeling to predict cognitive conversion based on a 4-point change in the AD assessment scale-cognition score. Treatment reassignment rates were estimated based on the actual and predicted cognitive conversion at 3 and 6 months according to the prespecified principle; that is if the ADAS-cog score of the patient declines less than 4 points or increases at either follow-up time point, the medical treatment recommended upon their diagnosis would be considered insufficient. Therefore, it is recommended to upgrade the medical treatment upon diagnosis. Actual and predicted treatment reassignment rates were compared in the general population and subpopulations categorized by age, gender, symptom severity, and the intervention subtypes.

**Results:**

A total of 224 patients were included in the analysis. The hybrid CNN-LSTM model achieved the mean AUC of 0.735 (95% CI: 0.701–0.769) at 3 months and 0.853 (95% CI: 0.814–0.892) at 6 months in predicting cognitive conversion status. The AUC at 6 months was significantly impacted when data collected at 3 months were withdrawn. The predicted cognitive conversion suggested a revision of medical treatment in 46.43% (104/224) of patients at 3 months and 54.02% (121/224) at 6 months as compared with 62.05% (139/224) at 3 months (*p* = 0.001) and 62.50% (140/224) at 6 months (*p* = 0.069) according to their actual cognitive conversion. No significant differences were detected between treatment reassignment rates estimated based on actual and predicted cognitive conversion in all directions at 6 months.

**Conclusion:**

Using the synergistic advances of deep learning modeling and featured longitudinal information, our hypothesis was preliminarily verified with the comparable predictive performance in cognitive conversion. Results provided the possibility of reassigned recommended treatment for those who may suffer from cognitive decline in the future. Considering the limited diversity of treatment strategies applied in this study, the real-world medical situation should be further simulated.

## Background

According to the Alzheimer’s Disease International, the estimated prevalence of dementia is about 50 million people worldwide in 2018 and will be projected to triple in 2050 (Koszewicz et al., 2021). Alzheimer’s disease (AD) and mild cognitive impairment (MCI) are two major types of dementia, which have launched significant economic load and medical burden for the families and healthcare systems (Hill et al., 2017).

With the increasing efforts in predicting the occurrence and development of AD and MCI, age and gender have often been identified as the most important risk factors. Previous studies have shown that for elders aged 65 years or more, there were significantly more women who have developed AD compared with a high proportion of men who have developed MCI with an OR of 1.54 (95%CI: 1.2–2.0) (Petersen et al., 2010; Wu et al., 2017). Moreover, studies on biomarkers have formed an important basis for the early diagnosis of dementia (Hort et al., 2010). The β amyloid deposition, pathologic tau, and neurodegeneration (A-T-N) framework proposed by Jack et al. (2018) is intended to define the diagnosis of AD by the presence of amyloid-β and phosphorylated tau that measured either in plasma, cerebrospinal fluid (CSF), or neuroimaging. While in the search for prognostic biomarkers of dementia, the field still focused heavily on neuroimaging and CSF markers. For instance, several studies have reported that the progression of AD or MCI could be predicted by advanced neuroimaging techniques, including MRI findings, FDG-PET, or CSF examination (Jack et al., 1999; Killiany et al., 2000; Shaw et al., 2009; Zhang et al., 2012). In fact, professionals have advocated that at least one neuroimaging examination and several CSF/plasma examinations are needed for monitoring the progression of dementia (Langa and Levine, 2014). However, the major barrier leading to this destination is the poor compliance to these examinations and the difficulties in the collection of the corresponding information in real clinical practice. In addition, even these difficulties can be conquered, their specificity and accuracy in the prediction of prognosis of dementia have not been guaranteed in the current context (Carrillo et al., 2013; Hepp et al., 2016).

For the majority of patients diagnosed with AD and MCI, cognitive impairment is recognized as the most dominant and patient-oriented symptom. Progression of cognitive impairment is normally monitored through longitudinal neuropsychological assessments using cognitive scales such as the Alzheimer’s Disease Assessment Scale-Cognition (ADAS-Cog) or the Mini-Mental State Examination (MMSE). This longitudinal information can be scheduled and collected sequentially through face-to-face interviews in the hospital, community, or at home. According to Xue et al. (2018) neuropsychological assessments were proved to be more practical compared with neuroimaging and CSF/plasma examinations and more accurate in the reflection of cognitive function than baseline information alone. This inspired us to ask whether sequential neuropsychological-related information along with other clinical data can be used to predict cognitive trajectory. Upon this thought, the long-term cognitive decline can be foreseen with which medical treatment for AD or MCI could be redirected at the early stage. Considering the high heterogeneity in the individual progression of dementia and huge difficulties in the prediction of its trajectory, traditional statistical models may not be powerful enough to provide accurate predictive outcomes. In particular, the cognitive trajectory that was impacted by the sequential time features would probably not be captured by traditional regression algorithms (Verbeke et al., 2014). Upon this data signature, we were, therefore, promoted to construct a novel deep learning model comprised of convolutional neural networks (CNN) and long-short-term memory (LSTM) networks. This hybrid CNN-LSTM network is capable to handle longitudinal data with varying lengths of follow-ups, help capture temporal dynamics, and, therefore, make accurate predictions based on the sequential data (Hochreiter and Schmidhuber, 1997; Cho et al., 2014; Chung et al., 2014; Ioannou et al., 2020).

In this study, we used the subset data from three ongoing, longitudinal, cohort studies conducted by our research team. The aim of this study was (1) to explore the predictive potential of longitudinal neuropsychological information on cognitive conversion reflected by ADAS-Cog upon the diagnosis of AD and MCI and (2) to compare the reassignment rates estimated by the deep learning modeling according to the actual status. If the long-term cognitive status can be successfully predicted with our hybrid CNN-LSTM modeling, then recommendations on treatment redirection can be provided at the early stage of AD or MCI.

## Materials and Methods

### Patient Data

**FIGURE 1:**
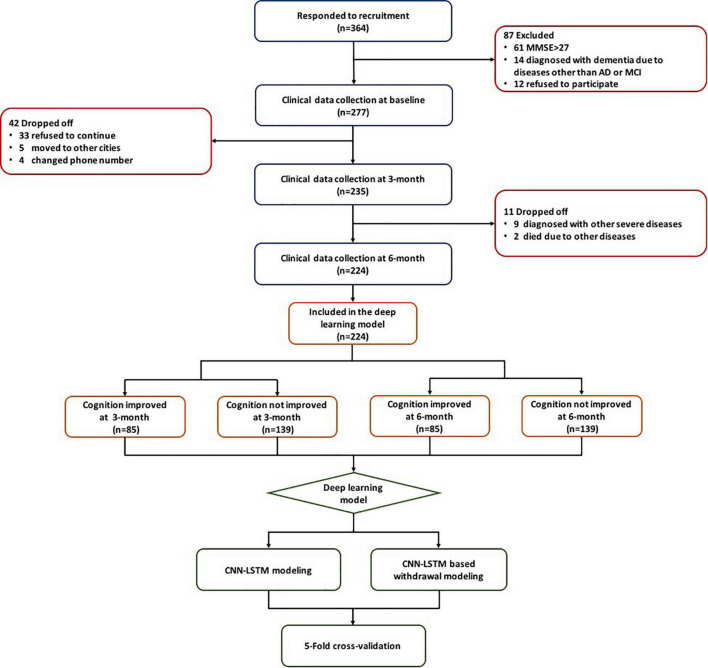
Flowchart of this study.

This study utilized three cohorts including 364 patients from the First Affiliated Hospital of Nanjing Medical University (ClinicalTrials.gov ID: NCT03090516 and Chinese Clinical Trial Registry ID: ChiCTR-INR-15007420) and 111 patients from communities in Nanjing, China. Patients from the above three cohorts were treated with either observation, exercise, monotherapy (donepezil or ginkgo biloba extract [GBE]), or donepezil and GBE combination. All of these patients met the following inclusion criteria: (1) diagnosed with AD or MCI according to the NINCDS/ADRDA guidelines; (2) MMSE score of 27 or less; (3) able to follow medical instruction or assessment requirement; (4) complete available baseline and longitudinal data, such as age, course of the disease, comorbidities, blood biochemical examination, clinical scales assessed during three visits (e.g., baseline, 3 months, and 6 months); and (5) signed informed consent (Cummings, 1993; Dubois et al., 2007). The flowchart of this study is shown in Figure 1.

### Predictive Variables and Target Outcomes

As data from these cohorts were presented with a varying treatment plan, disease duration, visiting frequency, and inclusion criteria, all patients have no data beyond 6 months at this moment. We chose a 3-month spacing between time points based on the visit frequency of follow-up patients to ensure most patients had efficient data for model building (baseline, 3 months, and 6 months).

Data in the merged database were stored in EXCEL format. The predictors used in our deep learning model of cognitive conversion prediction that were originated from tables in the database but not limited to demographics, medical history, intervention subtype, neuropsychological outcomes, and laboratory and neuroelectrophysiological results are listed in Supplementary Table 1. Time-dependent variables such as ADAS-cog (Rosen et al., 1984), MMSE (Folstein et al., 1975), the instrumental activity of daily living (IADL) (Thomas et al., 1998), geriatric depression scale (GDS) (Defrancesco et al., 2018), quality of life-Alzheimer’s disease (QOL-AD) (Lawton, 1997), and neuropsychiatric inventory (NPI) (Cummings et al., 1994) for the evaluation of neurobehavioral status were recorded at 3 month intervals (Cummings, 1993; Logsdon et al., 2002; LaPlante, 2010; Benedetti et al., 2018; Jiang et al., 2020; Vik-Mo et al., 2020).

According to the guidelines published by the American College of Physicians and the American Academy of Family Physicians, a decline of 4 points or more in the ADAS-cog score is used to define a clinically important improvement (Qaseem et al., 2008). Based on this, we used ADAS-cog as the target outcome and adopted 4 points as the threshold so that patients with different cognitive conversion during follow-ups were further classified (Qaseem et al., 2008; Raina et al., 2008). Specifically, patients whose ADAS-cog score had a decline of 4 points or more were grouped as cognition improved (CI), and patients whose ADAS-cog score remained stable or increased were grouped as cognition not improved (CNI) (Sabbagh et al., 2020).

### The Hybrid Convolutional Neural Networks and Long-Short-Term Memory Modeling

#### Data Preprocessing

We first excluded patients with missing feature variables covering more than 50% of the whole record number, as higher proportions of missing data limit the prediction ability of the proposed model. We then imputed missing data of remaining features with the mean or the mode of existing data in the same feature for continuous or categorical variables, respectively (Kang, 2013). Normalization was subsequently applied; therefore, all data are normalized to have zero mean and unit variance.

#### Model Development and Training Details

**FIGURE 2:**
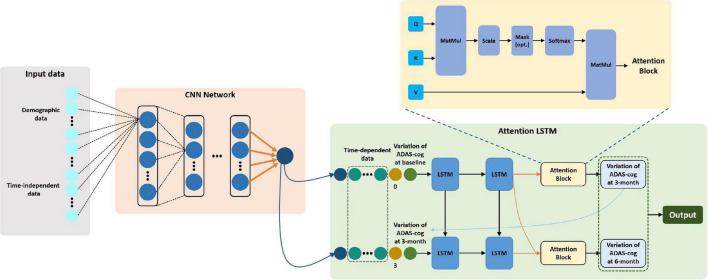
Workflow of CNN-LSTM modeling and architecture of proposed network composition. *The architecture of the hybrid CNN-LSTM model combines two modules. The CNN module (light pink) is composed of five fully connected layers and a rectified linear unit (ReLU) activation unit and then followed by a sigmoid activation function to generate a score representative of non-time dependent features. Next, the LSTM module (light green) was trained to extract both non- and time-dependent features to generate outputs for estimating cognitive conversion status in multiple time points. The attention block (light yellow) denoted an attention mechanism proposed by Vaswani et al. (2017) which was used to perform feature-weighted fusion across time steps to make an accurate prediction. *The yellow block used here refers to “scaled dot-product attention,” explicitly explained by Vaswani et al. (2017) Given a task-related query vector Q, it can calculate the attention value by calculating the attention distribution associated with the key (K) and assigning it to the value (V). This attention block is applied to find the “resonance” between hidden vectors from each time step, namely, find the most relevant embedding features for high-level representations recognition.

A hybrid model comprised of cascaded classical CNN and state-of-art LSTM was constructed to forecast 3-month interval cognitive conversion status (CI vs. CNI at 3 months and 6 months) in individual patients (Figure 2). Briefly, CNN with stacked multiple full connection layers was used to aggregate and extract features of all non-sequential state information. We then applied the sigmoid activation function to generate the score of the non-sequential state (time-independent data) information. The detailed architecture of CNN was explained in Supplementary Figure 1. Afterward, the generated score was combined with all other time-dependent data, the corresponding time-sequential information (baseline or 3-month) as well as the cognitive conversion status (improved or not) to fuse into the LSTM network. This specialized LSTM model was trained to use the encoded latent representation from the previous time point as well as the multivariate relations of the current time point (Gers and Schmidhuber, 2001). Using this process, the underlying temporal characteristics in the given time-dependent ADAS-cog score were captured. Notably, the attention mechanism was applied to perform feature-weighted fusion across time steps to improve the prediction accuracy (Vaswani et al., 2017). This procedure was made to empower the model to find significant useful information related to the current output in the input data and eventually improve the quality of the output.

We implemented the above model building on the publicly available library sklearn and open framework PyTorch on Python 3.6 using a computer with one NVidia GTX 1080Ti GPU. The batch size was set to 3,600 when training the CNN-LSTM model, with the optimizer used as the Adam algorithm (Kingma and Ba, 2014). The learning rate was reduced from 0.01, and the number of iterations of training is 1,000 epochs. The output model was selected based on the epoch, resulting in the highest AUC in the validation cohorts. For training the specialized LSTM model, the teacher forcing strategy is used to improve the learning efficiency of the model.

#### Model Withdrawal Procedure

To explore the impact of the cognitive conversion status at each time step during the evaluation of the patient, we used the trained hybrid model to test and evaluate the robustness of a single time point on the ADAS-cog score; that is, by changing the model input through withdrawing the mid-point information (simulating situations of follow-up absence), the degree of impact of ADAS-cog at 3 months can be evaluated.

### Model Performance Measurements and Statistical Analysis

Descriptive statistics (mean, standard deviation counts, and proportions) were provided to demonstrate the sample characteristics with respect to statistical quantitative and qualitative features.

For hybrid CNN-LSTM model evaluation, five-fold cross-validation was applied, and assembled ROC curves with AUCs were used to assess model performance at 3 and 6 months. Performance measurements including AUC, sensitivity, and specificity with 95% confidence interval (95% CI) were assessed using the optimal cut-off value (Youden index = sensitivity + specificity − 1).

We expected that the implementation of deep learning modeling could potentially guide the recommended treatment plan, especially on patients who may suffer from continuous cognitive decline during follow-ups. According to recommendations by multiple guidelines, it is assumed that if patient’s ADAS-cog score declines less than 4 points or increases at either follow-up time point (every 3 months), the medical treatment recommended upon their diagnosis would be considered insufficient for avoiding the cognitive decline in the future (Farlow and Cummings, 2007; Cummings et al., 2015; Grossberg et al., 2019). Therefore, it is recommended to redirect or upgrade the medical treatment. Upon the above rationales, it was hypothesized that if our deep learning modeling could successfully predict the long-term cognitive conversion, it could be used to guide the early decision-making redirection. Therefore, we first estimated the treatment reassignment rates based on the actual and predicted cognitive conversion (namely, the target outcome) at 3 and 6 months according to the above principle. The actual and predicted treatment reassignment rates were then compared with chi-square testing (adjusted if necessary) in the general population and according to age, gender, and symptom severity as well as intervention subtypes within all directions. STATA 16.0 (StataCorp LLC, TX, United States) was utilized to perform the above analysis, and the statistical significance was determined with *p* < 0.05.

## Results

### Demographics and Cognitive Conversion Distribution

Patient demographics and distribution according to cognitive conversion (e.g., improved or not improved) at 3 and 6 months are demonstrated in Table 1. A number of 224 patients were included, of whom 85 at 3 months and 84 at 6 months were identified as CI population, while 139 at 3 months and 140 at 6 months were identified as CNI. There were more men (137 accounted for 61.16%) than women (87 accounted for 38.84%) enrolled with an average age of 69.75 ± 8.52 years. Hypertension was the predominant comorbidity in 44 (19.64%) patients. Patients were referred for different treatments mainly based on the severity of cognitive impairment and the interests of the original cohorts in which they were enrolled, including 62 (27.68%) for observation, 22 (9.82%) for exercise, 55 (24.55%) for donepezil monotherapy, 38 (16.96%) of GBE monotherapy, and 47 (20.98%) for GBE and donepezil combination.

**TABLE 1:** Demographics and cognitive conversion distribution at 3 and 6 months.

Patient characteristics	Total (*n* = 224)	3-month		6-month	
		Cognition not improved (*n* = 139)	Cognition improved (*n* = 85)	Cognition not improved (*n* = 140)	Cognition improved (*n* = 84)
**Age in year, mean (SD)**	69.75 (8.52)	69.24 (9.17)	70.58 (7.32)	69.13 (9.07)	70.79 (7.45)
**Gender, n (%)**					
Male	87 (38.84)	51 (36.69)	36 (42.35)	52 (37.14)	35 (47.67)
Female	137 (61.16)	88 (63.31)	49 (57.65)	88 (62.86)	49 (58.33)
Education in year, mean (SD)	13.00 (4.32)	13.22 (4.72)	12.66 (3.55)	13.31 (4.79)	12.49 (3.35)
Height in centimeter	162.70 (3.69)	162.71 (4.29)	162.67 (2.44)	162.61 (3.92)	162.84 (3.30)
Weight in kilogram	63.09 (4.98)	62.72 (4.41)	63.71 (5.77)	62.58 (4.19)	63.95 (6.00)
**Comorbidities, n (%)**					
Hypertension	44 (19.64)	27 (19.42)	17 (20.00)	28 (20.00)	16 (19.05)
Diabetes mellitus	16 (7.14)	10 (7.19)	6 (7.06)	8 (5.71)	8 (9.52)
Thyropathy	6 (2.68)	5 (3.60)	1 (1.18)	6 (4.29)	0 (0.00)
Cardiovascular disorders	13 (5.80)	7 (5.04)	6 (7.06)	9 (6.43)	4 (4.76)
Asthma	3 (1.34)	1 (0.72)	2 (2.35)	1 (0.71)	2 (2.38)
Cerebrovascular disorders	17 (7.59)	14 (10.07)	3 (3.53)	13 (9.29)	4 (4.76)
Hyperlithuria	1 (0.45)	1 (0.72)	0 (0.00)	0 (0.00)	1 (1.19)
Hyperlipidemia	7 (3.12)	5 (3.60)	2 (2.35)	5 (3.57)	2 (2.38)
**Interventions, n (%)**					
Observation	62 (27.68)	53 (38.13)	9 (10.59)	56 (40.00)	6 (7.14)
Exercise	22 (9.82)	15 (10.79)	7 (8.24)	17 (12.14)	5 (5.95)
Donepezil	55 (24.55)	30 (21.58)	25 (29.41)	26 (18.57)	29 (34.52)
GBE	38 (16.96)	16 (11.51)	22 (25.88)	17 (12.14)	21 (25.00)
Donepezil and GBE	47 (20.98)	25 (17.99)	22 (25.88)	24 (17.14)	23 (27.38)
HIS, mean (SD)	0.97 (0.81)	1.01 (0.81)	0.91 (0.81)	0.99 (0.80)	0.95 (0.83)
**Family medical history, n (%)**					
Yes	45 (20.09)	26 (18.71)	19 (22.35)	24 (17.14)	21 (25.00)
No	179 (79.91)	113 (81.29)	66 (77.65)	116 (82.86)	63 (75.00)
MMSE, mean (SD)	23.46 (3.83)	23.96 (3.86)	22.64 (3.64)	23.94 (3.95)	22.65 (3.49)
ADAS-Cog, mean (SD)	16.62 (9.55)	13.79 (8.88)	21.25 (8.82)	13.97 (9.26)	21.04 (8.35)
IADL, mean (SD)	15.63 (2.47)	15.55 (2.38)	15.75 (2.62)	15.60 (2.34)	15.68 (2.69)
NPI, mean (SD)	3.47 (9.55)	3.17 (7.44)	3.97 (12.28)	4.11 (11.49)	2.41 (4.73)
QOL-AD, mean (SD)	31.91 (6.26)	32.53 (6.21)	30.89 (6.23)	32.33 (6.22)	31.21 (6.30)
GDS, mean (SD)	6.72 (6.44)	6.66 (6.33)	6.81 (6.65)	6.64 (6.07)	6.84 (7.04)
Anxiety, mean (SD)	1.25 (1.94)	0.79 (1.64)	2.01 (2.16)	0.84 (1.61)	1.95 (2.24)
CDR, mean (SD)	1.07 (0.27)	1.07 (0.29)	1.07 (0.26)	1.06 (0.26)	1.10 (0.30)
DSST, mean (SD)	31.86 (8.02)	32.27 (9.58)	31.18 (4.39)	31.99 (9.72)	31.63 (3.82)
TMT A, mean (SD)	82.88 (21.63)	82.08 (24.95)	84.18 (14.72)	83.08 (26.00)	82.53 (11.16)
TMT B, mean (SD)	218.28 (56.84)	217.75 (65.81)	219.14 (38.24)	217.94 (66.13)	218.84 (36.84)

### Predictive Performance of Hybrid Convolutional Neural Networks and Long-Short-Term Memory Modeling

**FIGURE 3:**
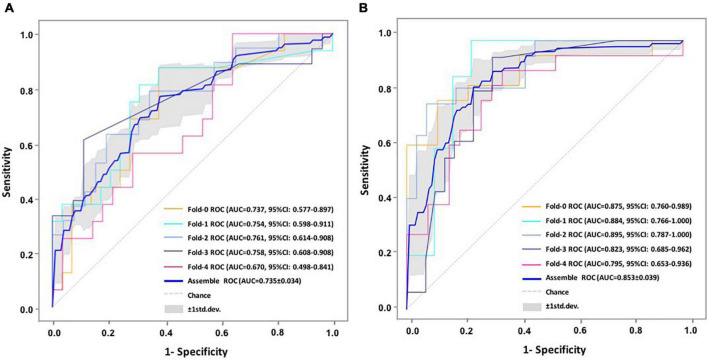
ROC comparisons of cognitive conversion at 3 and 6 months with CNN-LSTM modeling. **(A)** ROC comparisons of cognitive conversion at 3 months; **(B)** ROC comparisons of cognitive conversion at 6 months.

**FIGURE 4:**
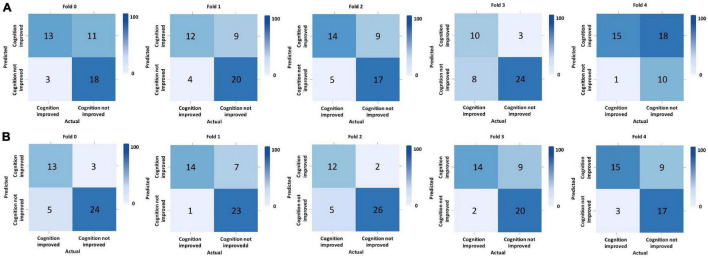
Predictive performance evaluation of CNN-LSTM modeling with confusion matrix at 3 and 6 months. *Computed classification confusion matrix using our hybrid CNN-LSTM modeling in five-fold cross-validation. **(A)** Confusion matrix at 3 months; **(B)** Confusion matrix at 6 months.

We performed five-fold cross-validation to test the model stability and to obtain the predicted outcomes of all patients. Our hybrid CNN-LSTM modeling showed a considerable good predictive capacity in identifying cognitive conversion status at sequential time points. As shown in Figure 3, the hybrid model achieved a mean AUC of 0.735 (95% CI: 0.701–0.769) at 3 months and 0.853 (95% CI: 0.814–0.892) at 6 months. The predictive performance achieved by the proposed model was considered good at 3 and 6 months, with the detailed information provided in Figure 4 and Table 2.

**TABLE 2:** Predictive performance evaluation of CNN-LSTM modeling at 3 and 6 months.

		Accuracy (95%CI)	Sensitivity (95%CI)	Specificity (95%CI)	Positive predictive value (95%CI)	Negative predictive value (95%CI)	F-score	AUC (95%CI)	AUPRC
Fold 0	3-month	0.711 (0.557–0.836)	0.875 (0.617–0.984)	0.621 (0.423–0.793)	0.560 (0.349–0.756)	0.900 (0.683–0.988)	0.683	0.737 (0.577–0.897)	0.522
	6-month	0.844 (0.705–0.935)	0.778 (0.524–0.936)	0.889 (0.708–0.976)	0.824 (0.566–0.962)	0.857 (0.673–0.96)	0.800	0.875 (0.760–0.989)	0.818
Fold 1	3-month	0.733 (0.581–0.854)	0.813 (0.544–0.960)	0.690 (0.492–0.847)	0.591 (0.364–0.793)	0.870 (0.664–0.972)	0.684	0.754 (0.598–0.911)	0.631
	6-month	0.844 (0.705–0.935)	1.000 (0.782–1.000)	0.767 (0.577–0.901)	0.682 (0.451–0.861)	1.000 (0.852–1.000)	0.811	0.884 (0.766–1.000)	0.666
Fold 2	3-month	0.711 (0.557–0.836)	0.790 (0.544–0.939)	0.654 (0.443–0.828)	0.625 (0.406–0.812)	0.810 (0.581–0.946)	0.698	0.761 (0.614–0.908)	0.680
	6-month	0.867 (0.732–0.949)	0.765 (0.501–0.932)	0.929 (0.765–0.991)	0.867 (0.595–0.983)	0.867 (0.693–0.962)	0.813	0.895 (0.787–1.000)	0.806
Fold 3	3-month	0.778 (0.629–0.888)	0.611 (0.357–0.827)	0.889 (0.708–0.976)	0.786 (0.492–0.953)	0.774 (0.589–0.904)	0.688	0.758 (0.608–0.908)	0.705
	6-month	0.778 (0.629–0.888)	0.938 (0.698–0.998)	0.690 (0.492–0.847)	0.625 (0.406–0.812)	0.952 (0.762–0.999)	0.750	0.823 (0.685–0.962)	0.571
Fold 4	3-month	0.591 (0.432–0.737)	1.00 (0.794–1.000)	0.357 (0.186–0.559)	0.471 (0.298–0.649)	1.000 (0.692–1.000)	0.640	0.670 (0.498–0.841)	0.479
	6-month	0.750 (0.597–0.868)	0.889 (0.653–0.986)	0.654 (0.443–0.828)	0.640 (0.425–0.820)	0.895 (0.669,0.987)	0.744	0.795 (0.653–0.936)	0.693

**FIGURE 5:**
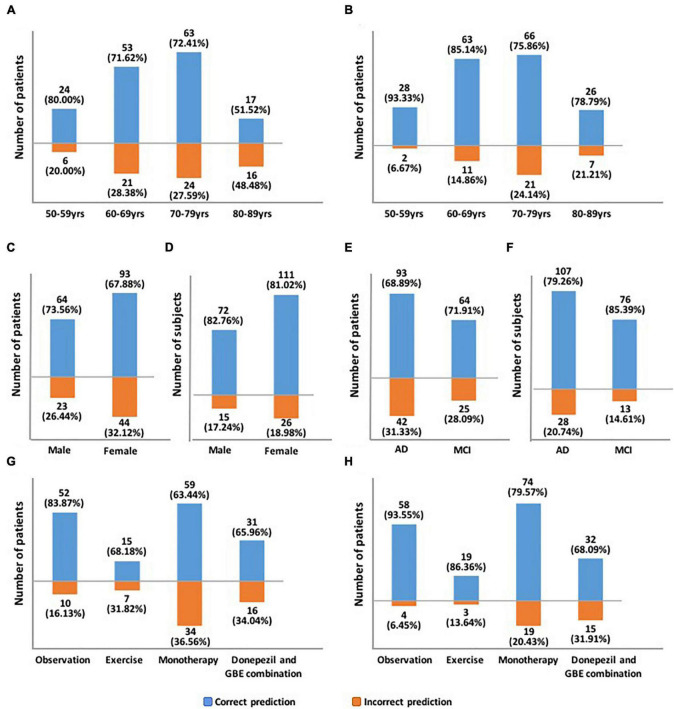
Demonstration of predictive accuracy stratified by age, gender, symptom severity, and intervention subtypes. **(A)** Predictive accuracy stratified by age at 3 months; **(B)** Predictive accuracy stratified by age at 6 months; **(C)** Predictive accuracy stratified by gender at 3 months; **(D)** Predictive accuracy stratified by gender at 6 months; **(E)** Predictive accuracy stratified by symptom severity at 3 months; **(F)** Predictive accuracy stratified by symptom severity at 6 months; **(G)** Predictive accuracy stratified by intervention subtypes at 3 months; **(H)** Predictive accuracy stratified by intervention subtypes at 6 months.

The proposed hybrid model generally provided accurately predicted outcomes in 70.09% of patients at 3 months and 81.70% at 6 months. To validate the generalizability of the proposed model, Figure 5 and Supplementary Table 2 provide the representative subgroup results according to age, gender, symptom severity, and intervention subtype between actual and predicted cognitive conversion at 3 and 6 months. Predictive performance was superior in male patients who were under observation at 3 months and exercise at 6 months.

In addition, we constructed the withdrawal model to further explore the importance of time-sequential data on the stability of our previously built CNN-LSTM modeling. As shown in Figure 3 and Supplementary Figure 2, without the incorporation of data collected at 3 months, the AUC at 6 months decreased from 0.853 (95% CI: 0.814–0.892) to 0.734 (95% CI: 0.678–0.790), indicating that the AUC was moderately impacted by the withdrawal of data collected at 3 months. Another evaluation matrix of CNN-LSTM based withdrawal modeling at 6 months is demonstrated in Supplementary Figure 3 and Table 3.

**TABLE 3:** Predictive performance evaluation of CNN-LSTM-based withdrawal modeling at 6 months.

	Accuracy (95%CI)	Sensitivity (95%CI)	Specificity (95%CI)	Positive predictive value (95%CI)	Negative predictive value (95%CI)	F-score	AUC (95%CI)	AUPRC
Fold 0	0.778 (0.629–0.888)	0.556 (0.308–0.785)	0.926 (0.757–0.991)	0.833 (0.516–0.979)	0.757 (0.577–0.889)	0.667	0.796 (0.656–0.937)	0.725
Fold 1	0.756 (0.605–0.871)	0.800 (0.519–0.957)	0.733 (0.541–0.877)	0.600 (0.361–0.809)	0.880 (0.688–0.975)	0.686	0.721 (0.554–0.888)	0.499
Fold 2	0.756 (0.605–0.871)	0.941 (0.713–0.999)	0.643 (0.441–0.814)	0.615 (0.406–0.798)	0.947 (0.740–0.999)	0.744	0.803 (0.661–0.944)	0.669
Fold 3	0.689 (0.534–0.818)	0.500 (0.247–0.753)	0.793 (0.603–0.920)	0.571 (0.289–0.823)	0.742 (0.554–0.881)	0.533	0.663 (0.491–0.834)	0.517
Fold 4	0.682 (0.524–0.814)	0.556 (0.308,0.785)	0.769 (0.564–0.910)	0.625 (0.354–0.848)	0.714 (0.513–0.868)	0.588	0.692 (0.529–0.855)	0.575

### Recommended Treatment Reassignment Following Actual and Predicted Cognitive Conversion at 3 and 6 Months

**FIGURE 6:**
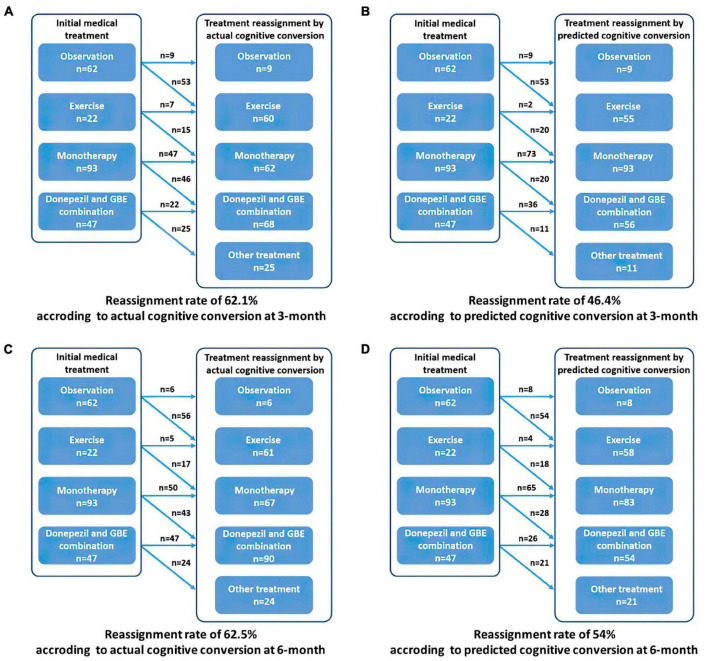
Recommended treatment reassignment following actual and predictive cognitive conversion at 3 and 6 months. **(A)** Treatment reassignment according to actual cognitive conversion at 3 months; **(B)** Treatment reassignment according to predicted cognitive conversion at 3 months; **(C)** Treatment reassignment according to actual cognitive conversion at 6 months; **(D)** Treatment reassignment according to predicted cognitive conversion at 6 months. *Another treatment indicates additional memantine, psychological interventions combined with pharmacological therapy or novel pharmacological approaches involving strategies to reduce amyloid and/or tau deposition.

Based on the prespecified principle, over half of the patients need reclassified treatment with a fraction of 62.05% (139/224) at 3 months and 62.50% (140/224) at 6 months as determined by their actual cognitive conversion. According to our hybrid CNN-LSTM algorithms, the predicted conversion could potentially guide the treatment reassignment in 46.43% (104/224) of patients at 3 months and 54.02% (121/224) at 6 months (Figure 6). No significant difference (*p* = 0.069) was noted in actual and predicted treatment reassignment at 6 months. We also estimated the treatment reassignment rate in subgroups categorized by age, gender, symptom severity, and intervention subtypes within all directions. Generally, no significant differences in treatment reassignment rates were detected between actual and predicted cognitive conversion in all directions at 6 months. Detailed statistics regarding subgroup analysis of treatment reassignment are provided in Table 4.

**TABLE 4:** Comparison between actual results and AI-predicted results after 3 and 6 months.

	3-month				6-month			
	Actual reassignment status	Predicted reassignment status	*X* ^2^	*p* value	Actual reassignment status	Predicted reassignment status	*X* ^2^	*p* value
**Overall, n (%)**	139/224 (62.05)	104/224 (46.43)	11.017	0.001	140/224 (62.50)	121/224 (54.02)	3.314	0.069
Observation	53/62 (85.48)	53/62 (85.48)	0.000	1.000	56/62 (90.32)	54/62 (87.10)	0.322	0.570
Exercise	15/22 (68.18)	20/22 (90.91)	–	0.132	17/22 (77.27)	18/22 (81.82)	–	1.000
Monotherapy	46/93 (49.46)	20/93 (21.51)	15.876	0.000	43/93 (46.24)	28/93 (30.11)	5.126	0.024
Donepezil and GBE combination	25/47 (53.19)	11/47 (23.40)	8.824	0.003	24/47 (51.06)	21/47 (44.68)	0.384	0.536
**Age, n (%)**								
**50**–**59y, n (%)**	25/30 (83.33)	23/30 (76.67)	0.147	0.519	24/30 (80.00)	24/30 (80.00)	0.000	1.000
Observation	15/17 (10.79)	16/17 (15.38)	–	1.000	15/17 (10.71)	15/17 (12.40)	–	1.000
Exercise	–	–	–	–	–	–	–	–
Monotherapy	7/10 (5.04)	5/10 (4.82)	–	0.650	7/10 (5.00)	6/10 (4.96)	–	1.000
Donepezil and GBE combination	3/3 (2.16)	2/3 (1.92)	–	1.000	2/3 (1.43)	3/3 (2.47)	–	1.000
**60**–**69yr, n (%)**	42/74 (56.76)	35/74 (47.30)	1.327	0.249	44/74 (59.50)	37/74 (50.00)	1.336	0.248
Observation	15/18 (83.33)	16/18 (88.89)	–	1.000	17/18 (94.44)	15/18 (83.33)	–	0.603
Exercise	6/8 (75.00)	8/8 (100.00)	–	0.467	6/8 (75.00)	7/8 (87.50)	–	1.000
Monotherapy	12/30 (40.00)	7/30 (23.33)	1.926	0.165	10/30 (33.33)	8/30 (26.67)	0.317	0.573
Donepezil and GBE combination	9/18 (50.00)	4/18 (22.22)	3.010	0.083	11/18 (61.11)	7/18 (38.89)	1.778	0.182
**70**–**79yr, n (%)**	49/87 (56.32)	33/87 (37.93)	5.905	0.015	51/87 (58.62)	42/87 (48.28)	1.871	0.171
Observation	16/19 (84.21)	15/19 (78.95)	–	1.000	17/19 (89.47)	17/19 (89.47)	–	1.000
Exercise	6/9 (66.67)	8/9 (88.89)	–	0.576	7/9 (77.78)	7/9 (77.78)	–	1.000
Monotherapy	19/39 (66.67)	7/39 (17.95)	8.308	0.004	18/39 (46.15)	10/39 (25.64)	3.566	0.059
Donepezil and GBE combination	8/20 (40.00)	3/20 (15.00)	–	0.155	9/20 (45.00)	8/20 (40.00)	0.102	0.749
**80**–**89yr, n (%)**	23/33 (69.79)	13/33 (39.39)	6.111	0.013	21/33 (63.64)	18/33 (54.55)	0.564	0.453
Observation	7/8 (87.50)	6/8 (75.00)	–	1.000	7/8 (87.50)	7/8 (87.50)	–	1.000
Exercise	3/5 (60.00)	4/5 (80.00)	–	1.000	4/5 (80.00)	4/5 (80.00)	–	1.000
Monotherapy	8/14 (57.14)	1/14 (7.14)	–	0.013	8/14 (57.14)	4/14 (28.57)	–	0.252
Donepezil and GBE combination	5/6 (83.33)	2/6 (33.33)	–	0.242	2/6 (33.33)	3/6 (50.00)	–	1.000
**Gender**								
**Male, n (%)**	51/87 (58.62)	40/87 (45.98)	2.778	0.095	52/87 (59.77)	45/87 (51.72)	1.142	0.285
Observation	21/25 (84.00)	22/25 (88.00)	–	1.000	23/25 (92.00)	21/25 (84.00)	–	0.667
Exercise	8/10 (80.00)	9/10 (90.00)	–	1.000	9/10 (90.00)	9/10 (90.00)	–	1.000
Monotherapy	14/35 (40.00)	6/35 (17.14)	4.480	0.034	15/35 (42.86)	8/35 (22.86)	3.173	0.075
Donepezil and GBE combination	8/17 (47.06)	3/17 (17.65)	–	0.141	5/17 (29.41)	7/17 (41.18)	0.515	0.473
**Female, n (%)**	88/137 (64.23)	64/137 (46.72)	8.511	0.004	88/137 (64.23)	76/137 (55.47)	2.187	0.139
Observation	32/37 (86.49)	31/37 (83.78)	0.107	0.744	33/37 (89.19)	33/37 (89.19)	–	1.000
Exercise	7/12 (58.33)	11/12 (91.67)	–	0.155	8/12 (66.67)	9/12 (75.00)	–	1.000
Monotherapy	32/58 (55.17)	14/58 (24.14)	11.672	0.001	28/58 (48.28)	20/58 (34.48)	2.275	0.132
Donepezil and GBE combination	17/30 (55.17)	8/30 (26.67)	5.554	0.018	19/30 (63.33)	14/30 (46.67)	1.684	0.194
**AD, n (%)**	76/135 (56.30)	56/135 (41.48)	5.929	0.015	78/135 (57.78)	66/135 (48.89)	2.143	0.143
**Symptom severity**								
Observation	24/31 (77.42)	23/31 (74.19)	0.088	0.767	27/31 (87.10)	25/31 (80.65)	–	0.731
Exercise	15/22 (68.18)	20/22 (90.91)	–	0.132	17/22 (77.27)	18/22 (81.82)	–	1.000
Monotherapy	21/52 (40.38)	9/52 (17.31)	6.746	0.009	20/52 (38.46)	11/52 (21.54)	3.722	0.054
Donepezil and GBE combination	16/30 (53.33)	4/30 (13.33)	–	0.002	14/30 (46.67)	12/30 (40.00)	0.271	0.602
**MCI, n (%)**	63/89 (70.79)	48/89 (53.93)	5.385	0.020	62/135 (45.93)	55/135 (40.74)	1.222	0.269
Observation	29/31 (93.55)	30/31 (96.77)	–	1.000	29/31 (93.55)	29/31 (93.55)	–	1.000
Exercise	–	–	–	–	–	–	–	–
Monotherapy	25/41 (60.98)	11/41 (26.83)	9.705	0.002	23/41 (56.10)	17/41 (41.46)	1.757	0.185
Donepezil and GBE combination	9/17 (52.94)	7/17 (41.18)	0.472	0.492	10/17 (58.82)	9/17 (52.94)	0.119	0.730

## Discussion

Progressive cognitive impairment is currently considered as the major symptom in patients with AD or MCI. Studies and reviews have suggested improved clinical outcomes (e.g., suspended or postponed cognitive decline) in patients who were managed upon early diagnosis (Livingston et al., 2017; Regan et al., 2017; Bachurin et al., 2018; Green et al., 2019; Mintun et al., 2021). Advanced diagnostic strategies (e.g., genetic or plasma screening) have been proposed, therefore early intervention could be delivered directly (Nakamura et al., 2018; Giau et al., 2019; Palmqvist et al., 2019; Karikari et al., 2020). However, there remain concerns regarding a fairly high proportion of the quick decline in cognitive function in a short period due to distinctions in terms of the progressive variation of dementia. In the view of medical professionals, this situation would be attributed to, to some extent, the lack of strategies available to guide treatment decision-making even the diagnosis of AD or MCI at the early stage.

The concept of artificial intelligence (AI) has recently permeated almost every sector of the healthcare system. Its recent expansion of plasma phosphor-tau (P-tau) and other biomarkers in the prediction regarding the risk of developing AD is an ideal example (Palmqvist et al., 2021). Nonetheless, there is an ongoing demand for the continual process of integrating and optimizing these synergistic advances in guiding medical decision-making in the real clinical context.

In this study, we first estimated the reassignment rates according to our prespecified principle (as mentioned earlier) using the datasets from three ongoing cohorts. Using AI techniques would allow us to compare the reassignment rates estimated by the deep learning modeling according to the actual status. Upon this perspective, our findings based on the predicted cognitive conversion at 3 and 6 months verified our hypothesis and provided preliminary recommendations on treatment reassignment at the early stage of AD or MCI.

**FIGURE 7:**
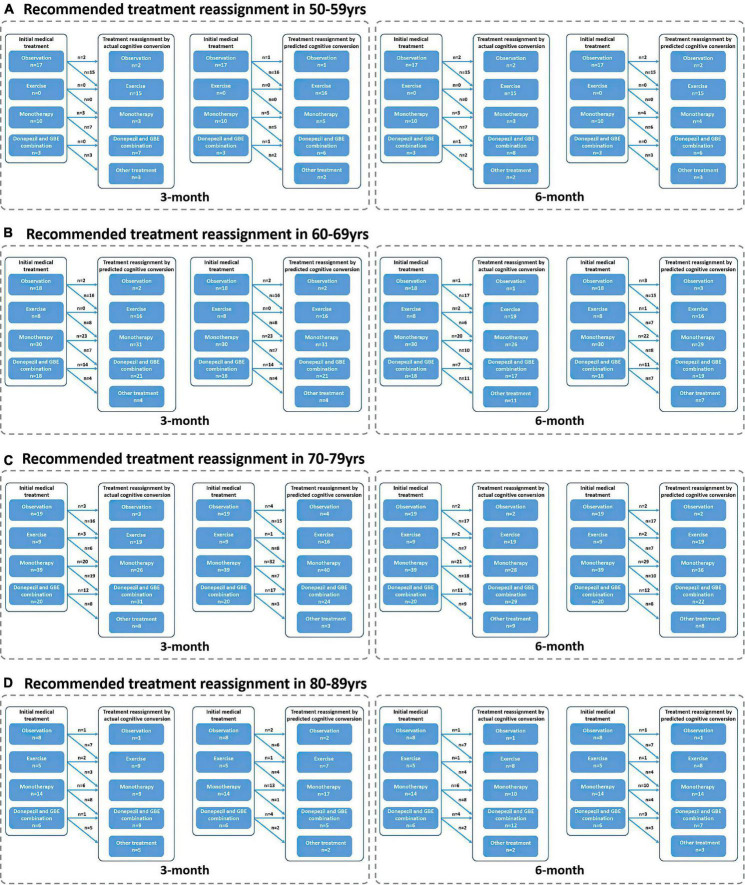
Recommended treatment reassignment following actual and predictive cognitive conversion according to age at 3 and 6 months. **(A)** Treatment reassignment according to actual and predicted cognitive conversion at 3 and 6 months in 50–59 years; **(B)** Treatment reassignment according to actual and predicted cognitive conversion at 3 and 6 months in 60–69 years; **(C)** Treatment reassignment according to actual and predicted cognitive conversion at 3 and 6 months in 70–79 years; **(D)** Treatment reassignment according to actual and predicted cognitive conversion at 3 and 6 months in 80–89 years. *Another treatment indicates additional memantine, psychological interventions combined with pharmacological therapy or novel pharmacological approaches involving strategies to reduce amyloid and/or tau deposition.

**FIGURE 8:**
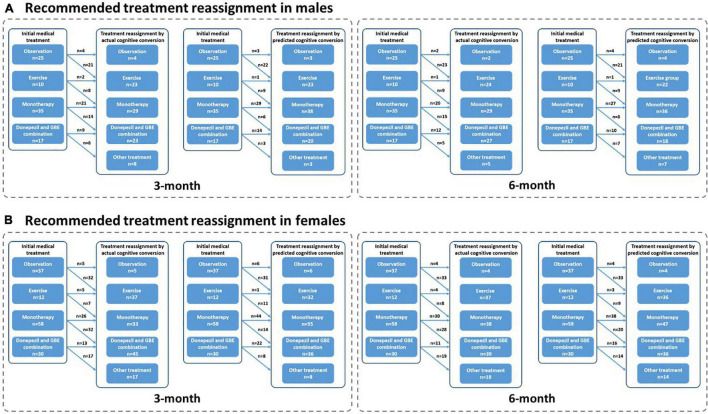
Recommended treatment reassignment following actual and predictive cognitive conversion according to gender at 3 and 6 months. **(A)** Treatment reassignment according to actual and predicted cognitive conversion at 3 and 6 months in men; **(B)** Treatment reassignment according to actual and predicted cognitive conversion at 3 and 6 months in women. *Another treatment indicates additional memantine, psychological interventions combined with pharmacological therapy or novel pharmacological approaches involving strategies to reduce amyloid and/or tau deposition.

**FIGURE 9:**
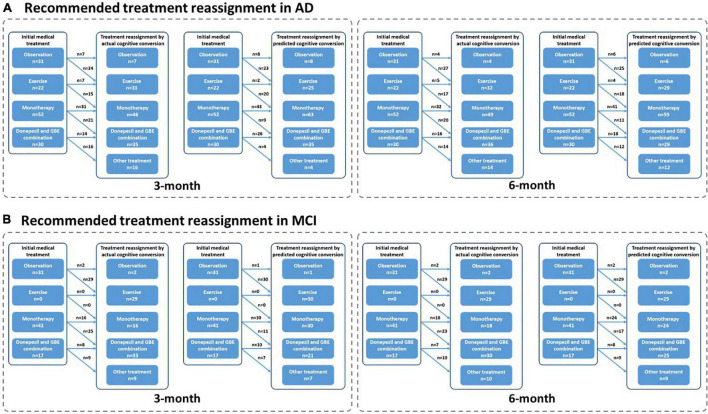
Recommended treatment reassignment following actual and predictive cognitive conversion according to symptom severity at 3 and 6 months. **(A)** Treatment reassignment according to actual and predicted cognitive conversion at 3 and 6 months in AD; **(B)** Treatment reassignment according to actual and predicted cognitive conversion at 3 and 6 months in MCI. *Another treatment indicates additional memantine, psychological interventions combined with pharmacological therapy or novel pharmacological approaches involving strategies to reduce amyloid and/or tau deposition.

Generally, predicted cognitive conversion according to our hybrid CNN-LSTM algorithms led to a recommendation of treatment reassignment in 46.43% (104/224) of patients at 3 months and 54.02% (121/224) at 6 months as compared with 62.05% (139/224) at 3 months (*X^2^* = 11.017, *p* = 0.001) and 62.50% (140/224) at 6 months (*X^2^* = 3.314, *p* = 0.069) according to their actual cognitive conversion (Figures 6–9 and Table 4). This indicated that a proportion of 74.7% of patients at 3 months and 86.4% at 6 months would be potentially benefited from deep learning modeling-guided treatment reassignment.

Interestingly, patients referred for observation and treated with monotherapy (GBE/donepezil) were more likely to be reclassified according to their actual cognitive conversion. However, a discrepancy that occurred between the actual and predicted reassignment status, with 44.4% (20/43) of patients at 3 months and 65.1% (28/46) at 6 months in this subgroup to be benefited, would be mainly attributed to the declined predictive accuracy in patients treated with monotherapy (GBE/donepezil). Considering the advantages of the hybrid CNN-LSTM algorithms in handling temporal data and capturing long-term dependencies, it was not surprising that the predictive performance was superior at 6 months than 3 months (Lara-Benítez et al., 2021). This was mainly benefited from its automatic feature learning ability with multiple temporal data (e.g., data at baseline and 3 months), which further enriched the predictive performance at 6 months. However, the predictive algorithm is different at 3 months, especially without the application of CNN featured algorithms. This highlighted the importance of adding time-sequential data to compensate for the above limitation in improving reassignment accuracy, while the increased costs and workload due to additional follow-ups would be further considered and balanced. It is important to emphasize that our follow-up is limited to 6 months at present, and long-term follow-up would be valuable to assess whether predictive performance can be further improved when the cognitive decline slows down with less heterogeneity and variation (Cortes et al., 2007; Vellas et al., 2007; Rockwood et al., 2008; De Rui et al., 2014). In contrast, the debated effectiveness of monotherapy (e.g., GBE and donepezil) may also increase the predictive uncertainty at both 3 and 6 months (Schneider et al., 2005; Mazza et al., 2006; Yuan et al., 2017; Birks and Harvey, 2018). Nonetheless, our results provided opportunities when neurological clinicians recommended medical treatment upon the diagnosis of AD or MCI, our model redirected management in averagely 80% of cases according to the actual cognitive conversion at 3 and 6 months, offering the potential to avoid a cognitive decline in the future. Unfortunately, a proportion of up to 26.3% of patients was not followed by our deep learning modeling, despite the dramatic efforts to optimize predictive performance and improve reassignment accuracy. Perhaps, this reflected nuanced medical management regarding factors such as age, gender, symptom severity, individual sensitivity, and variations in response to medical treatment, compliance to medical treatment, or other factors, which were not included in this study. Results of our subgroup analysis in reassignment rates also supported the above assumption.

Importantly, our findings could be placed into the context of guidelines highlighting the evidence-based classification criteria according to symptomatology and physiopathology to enable real-world testing of the value of our proposed strategy in a fashion that may assist the decision-making upon the diagnosis of AD or MCI. Since the diversity of medical treatment was limited due to the utilization of datasets from three registered cohorts, it cannot completely represent the real-world situation. However, our study may serve as a template and has preliminarily verified by the application of deep learning modeling-guided decision-making in the treatment of dementia among AD or MCI. In fact, the currently proposed deep learning modeling successfully simulated the actual reassignment status, and several concerns are pending for investigation. For instance, the prognosis following the deep learning modeling-guided reassignment of medical treatment needs to be verified with well-designed studies before its application in real clinical circumstances. We used the increase in ADAS-cog score for more than 4 points as the cut-off to define cognitive decline. Similar to all neuropsychological assessments, the actual cognitive function may not be completely followed due to the sensitivity and specificity of ADAS-cog (Chu et al., 2000; Monllau et al., 2007; Rockwood et al., 2007; Balsis et al., 2012; Skinner et al., 2012; Verma et al., 2015). Although we reported the actual and predicted reassignment rates according to our prespecified principles, it was not totally evidence-based, indicating that some elements of referral bias cannot be excluded in this study. Our deep learning modeling was featured with handling time-sequential data especially for longitudinal cognitive scores extracted from multiple neuropsychological assessments, and the lack of data in terms of Aβ, tau, and other neurodegenerative biomarkers would consequently be assumed to impact the predictive performance of our deep learning modeling (Choi and Jin, 2018; Lee et al., 2019; Janelidze et al., 2020). In fact, plasma and CSF samples were not planned in the three registered cohort studies, highlighting that future studies must take these elements into account.

## Conclusion

To sum up, we provided an example of hybrid CNN-LSTM modeling-driven early decision-tailoring in AD or MCI. Using deep learning modeling and featured longitudinal information, our hypothesis was preliminarily verified so that it may provide those suffering from cognitive decline in the future with chances to redirect treatment at the early stage. Considering the limited diversity of treatment strategies applied in this study, the real-world medical situation was not comprehensively simulated. Enlarged sample and diverse treatment datasets need to be further tested when considering the integration of this novel AI strategy into routine clinical practice of AD and MCI.

## Data Availability Statement

The original contributions presented in the study are included in the article/Supplementary Material, further inquiries can be directed to the corresponding authors.

## Ethics Statement

The studies involving human participants were reviewed and approved by the First Affiliated Hospital of Nanjing Medical University (Reference numbers: 2013-SRFA-089 and 2016-SR-134). The patients/participants provided their written informed consent to participate in this study.

## Author Contributions

TW and YZ conceived the design of the study and prepared the original draft. TW and QH contributed to data curation, funding acquisition, supervision, and critical revision of the manuscript. YZ and YL were responsible for designing the statistical strategy. JW, YX, SY, WL, and HS were responsible for data acquisition. All authors read and contributed intellectually important content and approved the final manuscript.

## Conflict of Interest

The authors declare that the research was conducted in the absence of any commercial or financial relationships that could be construed as a potential conflict of interest.

## Publisher’s Note

All claims expressed in this article are solely those of the authors and do not necessarily represent those of their affiliated organizations, or those of the publisher, the editors and the reviewers. Any product that may be evaluated in this article, or claim that may be made by its manufacturer, is not guaranteed or endorsed by the publisher.

## Abbreviations

AD: Alzheimer’s disease
MCI: mild cognitive impairment
CSF: cerebrospinal fluid
MRI: magnetic resonance imaging
FDG-PET: fluorodeoxyglucose positron emission tomography
ADAS-Cog: Alzheimer’s Disease Assessment Scale-Cognition
MMSE: Mini-Mental State Examination
GBE: ginkgo biloba extract
CNN: convolutional neural networks
LSTM: long-short-term memory
NINCDS-ADRDA: National Institute of Neurological and Communicative Disorders and Stroke and the Alzheimer’s Disease and Related Disorders Association
IADL: instrumental activity of daily living
GDS: geriatric depression scale
QOL-AD: quality of life-Alzheimer’s disease
NPI: neuropsychiatric inventory
CI: cognition improved
CNI: cognition not improved
HIS: Hachinski ischemic score
CDR: clinical dementia rating
DSST: digit symbol substitute test
TMT A: trail making test A
TMT B: trail making test B
ALT: alanine aminotransferase
TC: total cholesterol
TG: triglyceride
LDL: low density lipoprotein
ERP: event-related potentials
ROC: receiver operating curve
AUC: area under curve
CI: confidence interval
AUPRC: area under precision-recall curve
AI: artificial intelligence
P-tau: phosphor-tau
Aβ: beta-amyloid
