# Supplementary material for: Predicted Cognitive Conversion in Guiding Early Decision-Tailoring on Patients With Cognitive Impairment
Source: Front Aging Neurosci. 2022 Feb 2;13:813923. doi: 10.3389/fnagi.2021.813923 (PMC8847748; doi:10.3389/fnagi.2021.813923)
Supplement: Supplementary file 1 [file Data_Sheet_1.docx]

**Supplementary material**

**Table S1 Detailed input information for deep learning models**

| **Inputs of Model** |
| --- |
| **Baseline demographic and clinical variables** |
| Age |
| Gender |
| Education (year) |
| Height (cm) |
| Weight (kg) |
| Course (year) |
| Interventions |
| HIS at baseline |
| Anxiety at baseline |
| CDR at baseline |
| DSST at baseline |
| TMT A time at baseline |
| TMT B time at baseline |
| ALT at baseline |
| Medical history (Hypertension) |
| Medical history (Diabetes mellitus) |
| Medical history (Thyropathy) |
| Medical history (Cardiovascular disorder) |
| Medical history (Asthma) |
| Medical history (Cerebrovascular disorder) |
| Medical history (Hyperlithuria) |
| Medical history (Hyperlipemia) |
| Family history (Alzheimer's disease) |
| **Time-dependent variables** |
| MMSE at baseline |
| MMSE at 3-month |
| MMSE at 6-month |
| ADAS-cog at baseline |
| ADAS-cog at 3-month |
| ADAS-cog at 6-month |
| IADL at baseline |
| IADL at 3-month |
| IADL at 6-month |
| NPI at baseline |
| NPI at 3-month |
| NPI at 6-month |
| QOL-AD at baseline |
| QOL-AD at 3-month |
| QOL-AD at 6-month |
| GDS at baseline |
| GDS at 3-month |
| GDS at 6-month |
| TC at baseline |
| TC at 3-month |
| TC at 6-month |
| TG at baseline |
| TG at 3-month |
| TG at 6-month |
| LDL at baseline |
| LDL at 3-month |
| LDL at 6-month |
| Urea at baseline |
| Urea at 3-month |
| Urea at 6-month |
| ERP (N1 latency) at baseline |
| ERP (N1 latency) at 3-month |
| ERP (N1 latency) at 6-month |
| ERP (N1 base-to-peak) at baseline |
| ERP (N1 base-to-peak) at 3-month |
| ERP (N1 base-to-peak) at 6-month |
| ERP (N1 peak-to-peak) at baseline |
| ERP (N1 peak-to-peak) at 3-month |
| ERP (N1 peak-to-peak) at 6-month |
| ERP (P2 latency) at baseline |
| ERP (P2 latency) at 3-month |
| ERP (P2 latency) at 6-month |
| ERP (P2 base-to-peak) at baseline |
| ERP (P2 base-to-peak) at 3-month |
| ERP (P2 base-to-peak) at 6-month |
| ERP (P2 peak-to-peak) at baseline |
| ERP (P2 peak-to-peak) at 3-month |
| ERP (P2 peak-to-peak) at 6-month |
| ERP (N2 latency) at baseline |
| ERP (N2 latency) at 3-month |
| ERP (N2 latency) at 6-month |
| ERP (N2 base-to-peak) at baseline |
| ERP (N2 base-to-peak) at 3-month |
| ERP (N2 base-to-peak) at 6-month |
| ERP (N1 peak-to-peak) at baseline |
| ERP (N2 peak-to-peak) at 3-month |
| ERP (N2 peak-to-peak) at 6-month |
| ERP (P3 latency) at baseline |
| ERP (P3 latency) at 3-month |
| ERP (P3 latency) at 6-month |
| ERP (P3 base-to-peak) at baseline |
| ERP (P3 base-to-peak) at 3-month |
| ERP (P3 base-to-peak) at 6-month |
| ERP (P3 peak-to-peak) at baseline |
| ERP (P3 peak-to-peak) at 3-month |
| ERP (P3 peak-to-peak) at 6-month |

*MMSE: mini-mental state examination; ADAS-Cog: Alzheimer’s disease assessment scale-cognitive section; IADL: instrumental activity of daily living; NPI: neuropsychiatric inventory; QOL-AD: quality of life-Alzheimer’s disease; GDS: geriatric depression scale; HIS: hachiski ischemic score; CDR: clinical dementia rating; DSST: digit symbol substitute test; TMT A: trail making test A; TMT B: trail making test B; ALT: alanine aminotransferase; TC: total cholesterol; TG: triglyceride; LDL: low density lipoprotein; ERP: event related potentials.*

**TMT is a tool widely used to assess the ability of flexibly switch attention between competing task-set representation. The standardized version of TMT compromises two task components, TMT A and TMT B. TMT performance is typically indexed by total time to completion.*

**DSST is extensively used to assess the cognitive domains including visual motor speed and coordination, capacity for learning, attention, concentration, and short-term memory. The score of DSST is the number of consecutive digit-symbol pairs correctly completed within 2 minutes, and the total score is up to 135.*

**ERPs are the most commonly used tools to assess cognitive processing with high temporal resolution, mainly include N100, P200, N200 and P300. Among which, N200 and P300 are related to mental state and attention of people. Prolonged latency and decreased amplitudes (including base-to-peak value and peak-to-peak value) reflect cognition decline.*

**Table S2 Predictive accuracy estimation stratified by age, gender, symptom severity and intervention subtypes**

| **Subgroup** | **Total** | **3-month** | |  | **6-month** | |
| --- | --- | --- | --- | --- | --- | --- |
|  |  | **Correct prediction** | **Incorrect prediction** |  | **Correct prediction** | **Incorrect prediction** |
| **Age, n (%)** | | | | | | |
| 50-59yrs | 30 | 24/30 (80.00) | 6/30 (20.00) |  | 28/30 (93.33) | 2/30 (6.67) |
| 60-69yrs | 74 | 53/74 (71.62) | 21/74 (28.38) |  | 63/74 (85.14) | 11/74 (14.86) |
| 70-79yrs | 87 | 63/87 (72.41) | 24/87 (27.59) |  | 66/87 (75.86) | 21/87 (24.14) |
| 80-89yrs | 33 | 17/33 (51.52) | 16/33 (48.48) |  | 26/33 (78.79) | 7/33 (21.21) |
| **Gender, n (%)** | | | | | | |
| Male | 87 | 64/87 (73.56) | 23/87 (26.44) |  | 72/87 (82.76) | 15/87 (17.24) |
| Female | 137 | 93/137 (67.88) | 44/137 (32.12) |  | 111/137 (81.02) | 26/137 (19.98) |
| **Symptom severity, n (%)** | | | | | | |
| AD | 135 | 93/135 (68.89) | 42/135 (31.11) |  | 107/135 (79.26) | 28/135 (20.74) |
| MCI | 89 | 64/89 (71.91) | 25/89 (28.09) |  | 76/89 (85.39) | 13/89 (14.61) |
| **Interventions, n (%)** | | | | | | |
| Observation | 62 | 52/62 (83.87) | 10/62 (16.13) |  | 58/62 (93.55) | 4/62 (6.45) |
| Exercise | 22 | 15/22 (68.18) | 7/22 (31.82) |  | 19/22 (86.36) | 3/22 (13.64) |
| Monotherapy | 93 | 59/93 (63.44) | 34/93 (36.56) |  | 74/93 (79.57) | 19/93 (20.43) |
| Donepezil and GBEcombination | 47 | 31/47 (66.00) | 16/47 (34.00) |  | 32/47 (68.09) | 15/47 (31.91) |

*AD: Alzheimer’s disease; MCI: mild cognitive impairment; GBE: ginkgo biloba extract*

**Figure S1 Illustration of the feed forward neural network architecture of the proposed CNN network**

**
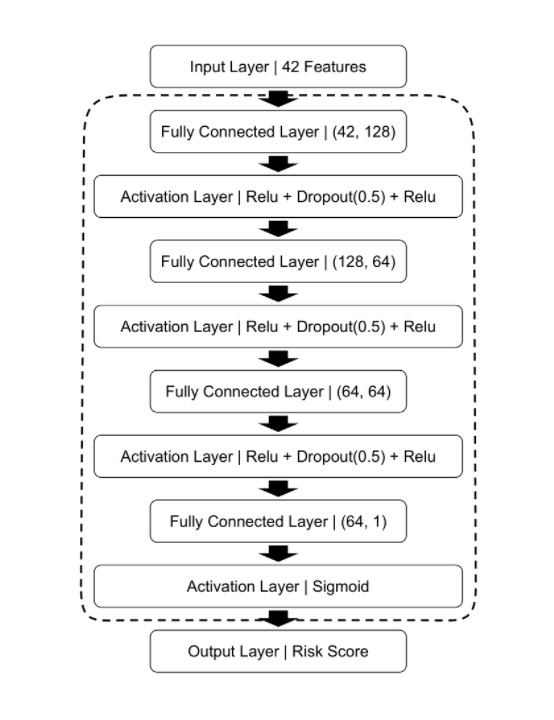
**

*CNN: convolutional neural networks.*

**Figure S2 ROC comparisons of cognitive conversion at 6-month with CNN-LSTM based withdrawal modeling**

**
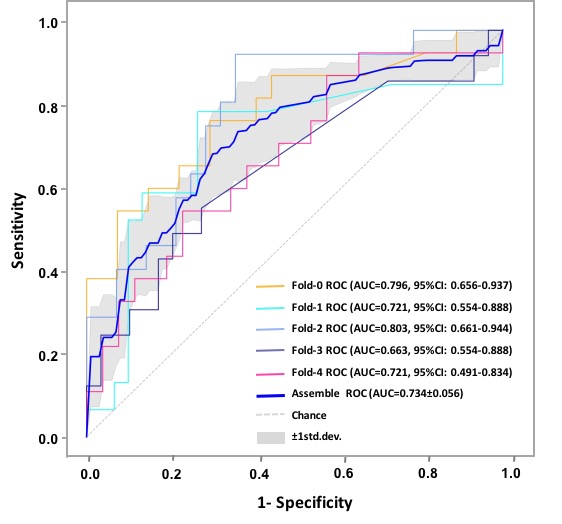
**

*ROC: receiver operating curve; AUC: area under curve; CI: confidence interval.*

**Figure S3** **Predictive performance evaluation of CNN-LSTM based withdrawal modeling with confusion matrix at 6-month**


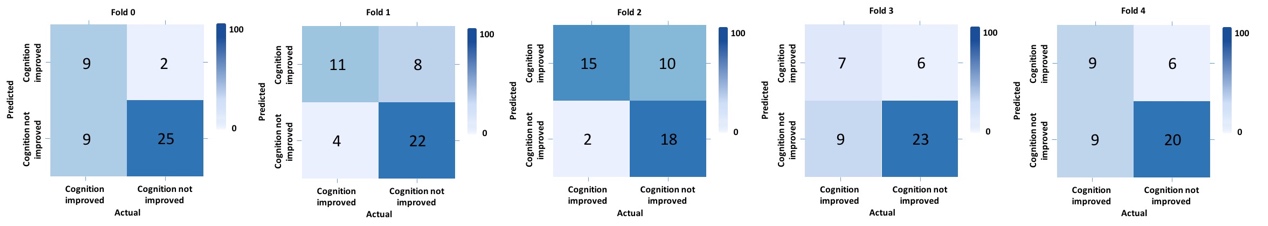


**Computed classification confusion matrix using our hybrid CNN-LSTM modelling in 5-fold cross-validation.*
